# The clinical advances of proteolysis targeting chimeras in oncology

**DOI:** 10.37349/etat.2021.00061

**Published:** 2021-12-31

**Authors:** Hao Xie, Junjia Liu, Diego M. Alem Glison, Jason B. Fleming

**Affiliations:** 1 Department of Gastrointestinal Oncology, H. Lee Moffitt Cancer Center & Research Institute, Tampa, FL 33612, USA; 2 Albert Einstein College of Medicine, Bronx, NY 10461, USA; University of Southampton, UK

**Keywords:** proteolysis targeting chimera, phase 1, clinical trial

## Abstract

Proteolysis targeting chimeras (PROTACs) are a class of small molecules designed to target proteins for degradation. Their novel and unique modes of action provide PROTACs with the potential for their application in the management of both solid and hematologic malignancies. Since its initial discovery, the technology of targeted protein degradation, especially in the form of PROTACs, has had significant advances. A number of PROTACs have entered a late stage of preclinical development. Several of them are either in phase 1/2 clinical trials or approaching approval for initial clinical evaluation. This article discusses the preclinical and clinical findings of PROTACs of clinically relevant protein targets in cancer.

## Introduction

The development of small molecule inhibitors in the setting of genomic profiling of the cancer (i.e., precision oncology) has dramatically transformed the management of both solid and hematologic malignancies in the past two decades. However, acquired resistance and/or cellular adaptation to small molecule inhibitors of oncoproteins and off-target toxicities have limited its clinical application to a certain extent [1]. As alternatives to small molecule inhibitors, targeted protein degradation in the form of proteolysis targeting chimeras (PROTACs) or molecular glues do not necessarily rely on direct binding to the proteins of interest at either catalytic or allosteric sites for their functions. Instead, they utilize the ubiquitin-proteasome system for targeted degradation of proteins of interest [2].

The distinct modes of action provide PROTACs with several advantages over traditional small molecule protein inhibitors (Table 1). First, PROTACs exhibit improved pharmacodynamic properties. In contrast to occupancy-driven pharmacology of small molecule inhibitors, the event-driven pharmacology enables PROTACs to function catalytically *in vivo*. As a result, PROTACs may be used at a lower dose to achieve comparable activity with much lower off-target toxicity [3]. Second, PROTACs have favorable pharmacokinetic properties. The time duration of the effect of small molecule inhibitors is largely dependent on the binding kinetics to their protein targets. Whereas PROTACs have been shown to maintain sustained target protein depletion with a single dose administration. Functional recovery of the target protein depends on the rate of protein resynthesis and the metabolism and excretion of PROTACs [4]. Third, PROTACs exhibit enhanced specificity for mutant target proteins and protein isoforms. This is likely due to favorable interactions between E3 complex and protein of interest mediated by optimal linker composition [5]. Lastly, PROTACs are less prone to drug resistance as a result of point mutations at the binding sites of small molecule inhibitors. PROTACs can tolerate low affinity binding with target proteins of interest to potentially bypass mutant sites [6]. For the same reason, PROTACs have the potential to target traditionally “undruggable” proteins by utilizing non-catalytic or non-allosteric surface binding sites or targeting kinase-independent protein functions or multi-protein complexes for degradation [7, 8].

**Table 1.** Clinically relevant advantages and limitations of PROTAC degraders compared to small molecule inhibitors

**Features**	**PROTAC degraders**	**Small molecule inhibitors**
Pharmacodynamic profile	Active in inhibitor-resistant cancer models [4, 44]	Acquired resistance is common [1, 44]
Selectivity	More selective and less off-target toxicity [3, 5, 45–47 ]	Side effects due to off-target toxicity [3]
Pharmacokinetic profile	Sustained target degradation and less frequent dosing [4]	Reversible target binding is common with frequent dosing [1]
Scope of application	Tolerate low affinity target binding for action; target protein complexes [6]	Require high affinity binding to protein target [6]
ADME	Higher molecular weight and potentially poor penetration to the cells; complex design may lead to rapid drug metabolism and excretion [4, 7]	ADME profile can be readily optimized [4]
Resistance mechanism	Complex design can be associated with multiple mechanisms of resistance [9]	Often due to point mutations at the binding pocket of protein target [4]

ADME: absorption, distribution, metabolism, and excretion

Despite many advantages of PROTACs over traditional small molecule inhibitors, several limitations are present that may restrict their further development as efficacious cancer therapeutics (Table 1). PROTACs as a class of heterobifunctional molecules often challenge Lipinski’s rule of five in drug discovery, which is often associated with poor cellular penetrations and adversely affected pharmacodynamic and pharmacokinetic properties [4]. In addition, the design of PROTACs consisting of binders of protein of interest, E3 ligase and a linker can often lead to off-target effects and undesirable issues with drug metabolism and excretion [7]. Furthermore, early acquired resistance has also been observed in cancer cells treated with PROTACs with the mechanisms attributed to molecular alterations of not only target protein of interest but also E3 ligase complexes [9].

The chemistry and chemical biology of PROTACs have been extensively reviewed elsewhere [10, 11]. This review article aims to summarize the preclinical and clinical findings of selected PROTACs that are in active clinical development for oncology to demonstrate if the advantages and limitations of RPOTACs are translated into the clinic (Table 2).

**Table 2.** Summary of ongoing clinical trials of PROTAC degraders

**Target**	**PROTAC name**	**Types of clinical trials**	**Patient population**	**ClinicalTrials.gov number**	**Sponsor**
AR	ARV-110	Phase 1/2	mCRPC	NCT03888612	Arvinas
AR	CC-94676	Phase 1	mCRPC	NCT04428788	Celgene/BMS
ER	ARV-471 alone or with palbociclib	Phase 1/2	ER^+^/HER2^−^ advanced or metastatic breast cancer	NCT04072952	Arvinas
BTK	NX-2127	Phase 1	Relapse/refractory B-cell malignancies	NCT04830137	Nurix Therapeutics
BCL-xL	DT2216	Phase 1	Relapse/refractory solid and hematologic malignancies	NCT04886622	Dialectic Therapeutics
BRD9	FHD-609	Phase 1	Advanced synovial sarcoma	NCT04965753	Foghorn Therapeutics

AR: androgen receptor; ER: estrogen receptor; BTK: Bruton’s tyrosine kinase; BCL-xL: B-cell lymphoma-extra large; BRD9: bromodomain-containing protein 9; mCRPC: metastatic castration resistant prostate cancer; HER2: human epidermal growth factor receptor 2

## Clinical trials of PROTAC degraders

### AR PROTAC

Androgen-deprivation therapy in combination with chemotherapy, enzalutamide, or abiraterone is the mainstay therapeutic strategy in patients with metastatic prostate cancer [12]. However, the disease eventually progresses to become castration-resistant. Yet, the cancer remains having high AR expression and dependent on intact AR signaling. Resistance to androgen-deprivation based therapy has been attributed to AR mutations, amplifications and autocrine tumor androgen production [12].

**Figure 1.**
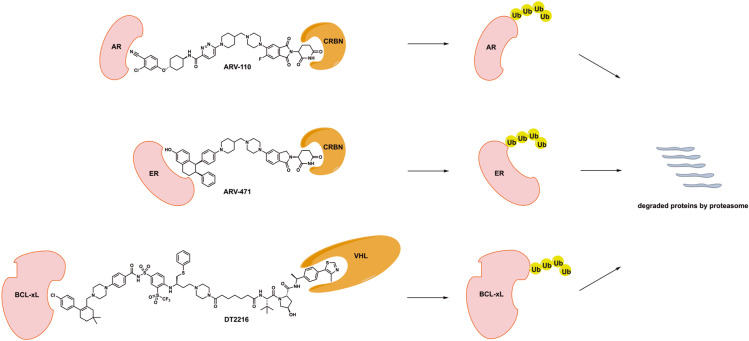
Chemical structures of disclosed PROTAC degraders in clinical development. CRBN: cereblon; Ub: ubiquitin; VHL: von Hippel-Lindau

ARV-110 was the first-in-class AR PROTAC developed by Arvinas (chemical structure shown in Figure 1) [13, 14]. *In vitro* evaluation demonstrated that ARV-110 efficiently degrades AR with half-maximal degradation concentration (DC_50 _) of approximately 1 nmol/L with marked selectivity in global proteomics analysis. ARV-110 inhibited the synthesis of prostate-specific antigen (PSA) and AR-dependent prostate cancer cell proliferation by inducing apoptosis. It demonstrated activity in enzalutamide refractory/resistant prostate cancer xenograft models with AR amplification and mutations with the exception of L702H mutation and AR-V7 variance. ARV-110 was able to reduce more than 90% of AR and suppress AR-associated gene expression *in vivo* [13, 14].

**Figure 2.**
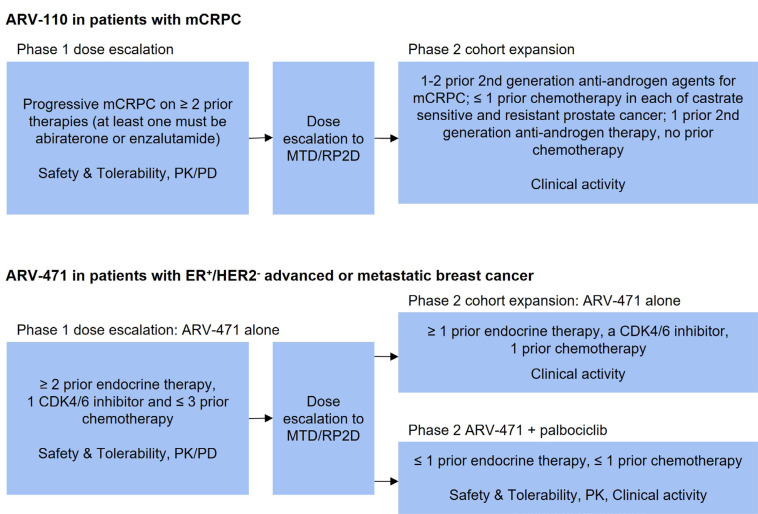
Design of selected clinical trials of PROTAC degraders. PK: pharmacokinetic; PD: pharmacodynamic; RP2D: recommended phase II dose; CDK4/6: cyclin dependent kinase 4/6

ARV-110 was the first AR PROTAC that entered the clinic. The clinical safety, tolerability and initial activity have been evaluated in the ongoing phase 1/2, open-label, dose escalation, and cohort expansion trial in patients with mCRPC whose disease is refractory to enzalutamide and/or abiraterone and progressed after at least two lines of previous therapy (NCT03888612) [15]. It is a single arm study with ARV-110 given orally, once daily in 28-day cycles. The study design is the traditional 3 + 3 design for dose escalation (Figure 2). Primary endpoints include maximal tolerated dose (MTD), incidence of dose limiting toxicities (DLT), adverse events for dose escalation phase, PSA and radiographic responses for dose expansion phase. Secondary endpoints include pharmacokinetic parameters and pharmacodynamic biomarker analysis as well as overall response, duration of response, progression-free survival (PFS), and overall survival (OS) [15].

The estimated goal of enrollment was 150 patients. At the time of initial abstract presentation, 22 patients received ARV-110 at different dose levels with maximal dose of 280 mg. One patient had a grade 4 transaminitis followed by acute renal failure as DLT. It was found that concurrent use of rosuvastatin and ARV-110 was associated with reversible grade 3/4 transaminitis in two patients due to significantly increased plasma concentration of rosuvastatin. Pharmacokinetic analysis demonstrated that the half-life of ARV-110 was approximately 110 h and plasma concentration associated with preclinical antitumor activity was reached at the 140 mg dose level. Significant reduction of AR protein expression has been observed in paired tumor specimens by immunohistochemistry before and after 6 weeks of treatment with ARV-110 at 280 mg. Two of the 22 evaluable patients had a durable PSA decline of greater than 50% with one of them having partial response (PR) defined by response evaluation criteria in solid tumors (RECIST) criteria. Last but not least, AR T878A and H875Y mutations, although associated with resistance to enzalutamide and abiraterone, were associated with better sensitivity to ARV-110 [13, 16].

In a subsequent press release, ARV-110 420 mg daily was selected as the recommended dose for phase 2 expansion cohort [17]. The phase 2 study aimed to enrich patients whose tumors carry AR T878 and H875 mutations and those who were less heavily pretreated, thus potentially more sensitive to ARV-110. In addition, ARV-766 and another AR PROTAC different from ARV-110, will enter phase 1 clinical trial for patients with metastatic prostate cancer soon [17].

CC-94676 (AR-LDD) is yet another AR PROTAC, initially developed by Celgene and now Bristol Myers Squibb (BMS) [18]. It has similar preclinical activity to ARV-110 in regards to effective AR protein degradation, favorable pharmacokinetic properties, and sustained suppression of tumor growth in VCAP CRPC mouse models [18]. It is currently in a phase 1 trial to evaluate its safety, tolerability, pharmacokinetics, and pharmacodynamics properties in patients with mCRPC whose disease progressed on androgen-deprivation therapy and at least one prior therapy such as abiraterone, enzalutamide or agents in the same class (NCT04428788) [19]. Yet, CC-94676 was not compared directly with ARV-110 for activities in the preclinical or clinical studies.

### ER PROTAC

Majority of newly diagnosed metastatic breast cancer cases are ER^+^, which serves as the major driver for disease progression. Agents targeting ER signaling including fulvestrant as a selective ER degrader have demonstrated significant clinical activity and efficacy. However, ER^+^, HER2^−^ metastatic breast cancer inevitably develops resistance to different types of ER-targeting agents [20]. ARV-471, an oral PROTAC degrader developed by Arvinas, targets ER for effective degradation (chemical structure shown in Figure 1).

In preclinical studies, ARV-471 degraded ERα in multiple ER^+^ breast cancer cell lines with DC_50_ of 0.9 nmol/L in MCF7 breast cancer cells [21]. In addition, ARV-471 robustly degraded mutant ERα Y537S and D538G which led to inhibition of downstream gene expression and decreased proliferation of ER-dependent breast cancer cell lines. ARV-471 also demonstrated *in vivo* activity in immature rat uterotrophic model, MCF7/E2 xenograft model, tamoxifen-resistant MCF7 mouse model, and ESR1 Y537S PDX model. ARV-471 had improved *in vivo* activity compared to fulvestrant and this activity was augmented by its combination with CDK4/6 inhibitors such as palbociclib [21].

The safety, tolerability, and clinical activity of ARV-471 alone and in combination with palbociclib have been evaluated in the ongoing phase 1/2 dose escalation and cohort expansion study (NCT04072952) in patients with ER^+^, HER2^−^, advanced or metastatic breast cancer who have received chemotherapy or hormonal therapy previously (Figure 2) [22]. The phase 1 part of this study utilized traditional 3 + 3 dose escalation with ARV-471 administered orally, once daily for 28-day cycles. The starting dose of ARV-471 was 30 mg. The primary endpoint of phase 1 part was to determine the MTD and recommended phase 2 dose. The secondary endpoints included adverse events, pharmacokinetic parameters and pharmacodynamic markers such as ER expression in paired biopsy samples before and after ARV-471 treatment. The activity endpoints included clinical benefit rate defined as complete response, PR, and stable disease longer than 24 weeks as determined by RECIST criteria [22].

As of November 2020, twenty-one patients were enrolled in the phase 1 part who were heavily pre-treated and thus carried poorer prognosis [22, 23]. Among them, 48% had visceral metastatic disease often in the liver and lung; 100% had previous CDK4/6 inhibitors; 71% had fulvestrant; 38% had chemotherapy; and 24% had other selective ER degraders in the clinical trial setting. They had a median of 5 previous lines of therapy for their advanced or metastatic breast cancer. ARV-471 was very well tolerated even up to the highest dose level of 360 mg with no grade 3/4 adverse events. No DLT has been reported and the MTD has not been reached. Pharmacokinetic study was informative that ARV-471 exposure at 60 mg daily dose or above exceeded the efficacious level in preclinical models with half-life of approximately 28 h. In paired tumor biopsy samples before and after treatment with ARV-471, mean ER degradation by quantitative immunofluorescence was 62% at all dose levels, significantly higher than 50% by fulvestrant. ER degradation has been observed in patients with either wild-type or mutant ER Y537S, Y537N, and D538G in their tumors. ARV-471 provided clinical benefit in 5 (42%) of the 12 evaluable patients for activity with 1 PR and 4 stable disease longer than 24 weeks. Compared to other selective ER degraders that are under active early phase clinical evaluations, ARV-471 provided the highest mean ER degradation and clinical benefit rate with the least incidence of severe toxicities. ARV-471 is currently under phase 2 development and its combination with palbociclib is also in phase 1b study [22, 23].

### BTK PROTAC

BTK plays an important role in the differentiation, proliferation, and survival of malignant B cells in different types of B-cell lymphomas and chronic lymphocytic leukemia (CLL). Ibrutinib and acalabrutinib are FDA-approved covalent BTK inhibitors that are clinically efficacious in the management of B-cell malignancies [24]. However, acquired resistance to these BTK inhibitors inevitably arises and leads to disease progression, with mechanisms attributed to acquired mutations and loss of covalent bond formation with the drug at cysteine 481 [25]. In addition, immunomodulatory imides such as lenalidomide have also demonstrated activity and efficacy in some types of B-cell malignancies as a result of neosubstrate degradation of transcription factors Ikaros family zinc finger protein 1/3 (IKZF1/3) [26].

NX-2127, developed by Nurix Therapeutics, is not only a BTK PROTAC degrader but also a molecular glue for IKZF1/3 degradation given it utilizes cereblon binder to recruit ubiquitin ligase complex for BTK degradation [27]. Degradation of two clinically validated targets simultaneously provided NX-2127 with the ability to potentially overcome resistance to existing BTK inhibitors. In the preclinical studies, NX-2127 degraded BTK effectively in both mantle cell lymphoma and diffuse large B-cell lymphoma (DLBCL) cells with DC_50_ of less than 5 nmol/L. In addition, NX-2127 robustly inhibited DLBCL cell proliferation that was resistant to ibrutinib and pomalidomide due to BTK C481S mutation with half-maximal effective concentration (EC_50 _) less than 30 nmol/L. Unlike clinically significant inhibition of platelet function by BTK inhibitors, NX-2127 did not show significant platelet inhibition in collagen-induced or collagen-related peptide-induced platelet aggregation assays *in vitro*. As expected from the PROTAC design of NX-2127, it induced IKZF3 degradation with similar DC_50_ of 25 nmol/L compared to lenalidomide and pomalidomide in naive human T cells, which led to IL-2 production and T cell activation following CD3/CD28 stimulation. Last but not least, NX-2127 demonstrated superior *in vivo* activity to ibrutinib in xenograft mouse models with wildtype BTK or mutant BTK C481S. In the cynomolgus monkey model, oral NX-2127 at 1 mg/kg daily brought sustained BTK degradation of greater than 90% despite its short half-life of 5.4 h [27].

The clinical safety, tolerability and activity of NX-2127 are currently evaluated in a first-in-human phase 1a/b study in patients with relapsed and refractory B-cell malignancies, whose disease progressed after at least 2 prior lines of therapy (NCT04830137) [28]. After initially dose escalation in phase 1a study, dose expansion of NX-2127 will be performed in 5 arms of phase 1b study. The 5 arms will include patients with or without BTK C481 mutation in their CLL/small lymphocytic lymphoma (SLL) which failed a BTK inhibitor, patients with mantle cell lymphoma, marginal zone lymphoma, and Waldenstrom macroglobulinemia which failed a BTK inhibitor and an anti-CD20 monoclonal antibody therapy, patients with DLBCL which failed an anthracycline and an anti-CD20 monoclonal antibody therapy, and patients with follicular lymphoma which failed an anti-CD20 monoclonal antibody therapy. The primary endpoints are DLT, recommended phase 1b dose for phase 1a study, overall response rate for phase 1b study, and adverse events in both studies. The secondary endpoints include pharmacokinetic parameters, complete response rate, duration of response, PFS, and OS [28].

### BCL-xL PROTAC

It has been very well established that the B-cell lymphoma 2 (BCL-2) family, including BCL-2, BCL-xL and myeloid cell leukemia 1, are involved in cell death control and apoptosis pathways as well as resistance to traditional chemotherapy agents in many cancers [29]. Small molecule BCL-xL inhibitors have been extensively evaluated as a therapeutic strategy to induce cancer cell apoptosis. However, some BCL-xL inhibitors suffered from on-target and dose-limiting thrombocytopenia which limited their clinical applications [30].

DT2216, developed by Dialectic Therapeutics, is a BCL-xL PROTAC degrader with its design utilizing a dual BCL-2/BCL-xL inhibitor ABT263 and a VHL E3 ligase binder (chemical structure shown in Figure 1) [31, 32]. DT2216 with robust BCL-xL degradation demonstrated superior potency to ABT263 in various BCL-xL-dependent cancer cells and yet much less toxicity to platelets due to lack of significant VHL expression in platelets. In addition, DT2216 exhibited synergy with other BCL-2 family inhibitors as well as chemotherapeutic agents. DT2216 has also demonstrated anticancer activity in several xenograft mouse models of both solid and hematologic malignancies without significant thrombocytopenia [31, 32]. For example, DT2216 caused significantly more reduction of viability of MyLa T-cell lymphoma cells (EC_50_ < 10 nmol/L) than ABT263, but was less toxic to platelets (EC_50_ > 3 μmo/L) than ABT263 [33]. DT2216 was able to effectively suppress BCL-xL expression and promote apoptosis in T-cell lymphoma xenograft models. This was translated into rapid tumor regression and improved survival of mice bearing T-cell lymphoma resistant to ABT263 when DT2216 was given at 10 mg/kg every 4 days. In addition, the activity of DT2216 was also observed in a similar fashion in T-cell acute lymphocytic leukemia patient-derived xenograft models [34]. More recently, BCL-xL degradation strategy using DT2216 has led to induced apoptosis of tumor-infiltrating regulatory T cells with effective tumor suppression in immunocompetent mice. This finding suggested that DT2216 may have the potential to be used as cancer immunotherapy [35].

With these promising preclinical findings, DT2216 is under clinical evaluation in a phase 1, open-label, dose escalation and cohort expansion study for its safety, tolerability, and activity in patients with relapse and refractory solid tumors and hematologic malignancies (NCT04886622). In this study, DT2216 is administered intravenously twice weekly in 28-day cycles for up to 12 months. The primary endpoints are DLT, adverse events and to find the recommended phase 2 dose. The secondary endpoints include pharmacokinetic profile, level of BCL-xL in peripheral mononuclear cells, platelet counts, and anticancer activity [36].

### BRD9 PROTAC

Patients with advanced synovial sarcoma benefit from very limited therapeutic options with median OS of only 18 months. The oncogenic fusion of SS18-SSX in synovial sarcoma renders its dependency on the activity of BRD9, a member of chromatin remodeling protein complex that orchestrates gene expression [37, 38]. CFT8634, developed by C4 Therapeutics, as a BRD9 PROTAC is superior to existing BRD inhibitors due to its high specificity towards BRD9 degradation over BRD4/7. As a result, CFT8634 may have improved toxicity profile compared to existing BRD inhibitors. In addition, CFT8634 showed excellent *in vivo* activity of tumor growth suppression in both cell-line and patient-derived xenograft models of synovial sarcoma [39].

FHD-609 is another BRD9 PROTAC degrader developed by Foghorn Therapeutics [40]. It is currently under clinical evaluation in a phase 1, open-label, dose escalation and expansion trial in patients with advanced synovial sarcoma (NCT04965753). FHD-609 is administered intravenously every 2 weeks. The primary endpoints are DLT and adverse events. The secondary endpoints include pharmacokinetic profile, clinical activities in the form of objective response rate, duration of response, time to response, PFS, and OS [40].

### Other PROTACs

Other PROTACs that are in late preclinical development and ready for investigational new drug (IND) application submission include KT-333, a signal transducer and activator of transcription 3 (STAT3) PROTAC degrader developed by Kymera Therapeutics, which demonstrated robust *in vivo* activity in peripheral T-cell lymphoma [41, 42], KT-413, a PROTAC degrader that selectively degrades both interleukin-1 receptor-associated kinase 4 (IRAK4) and IKZF1/3 also developed by Kymera Therapeutics, which has *in vivo* activity in myeloid differentiation primary response 88 (MyD88)-mutant DLBCL xenograft models [43], and CG001419, a tropomyosin receptor kinase (TRK) PROTAC degrader developed by Cullgen.

## Conclusions

PROTACs have demonstrated excellent preclinical activities in various types of cancers by robust and specific target protein degradation. Some of these preclinical activities were also present in *in vivo* cancer models that were resistant to the counterpart small molecule inhibitors of PROTACs. These findings have been translated into clinical activities observed in phase 1 clinical trials of AR and ER PROTACs in patients with advanced and refractory prostate or ER^+^/HER2^−^ breast cancer, respectively. More importantly, these PROTAC degraders with limited off-target toxicities were very well tolerated by heavily pretreated patients. In addition, clinical PROTAC degraders of AR and ER as reported had favorable pharmacokinetics profiles despite initial concern based on their violation of Lipinski’s rule of five. As a novel class of small molecule therapeutics in oncology, PROTAC degraders may start entering their prime time given these initial promising clinical observations. Future clinical development of PROTAC degraders should focus more on the evaluation of their clinical efficacy and adverse events in larger clinical trials, exploration of potential resistance mechanisms, synergy with other cancer therapeutic agents, and the development of companion biomarkers for optimal patient selection in future clinical trials.

## Abbreviations

AR: androgen receptor
BCL-2: B-cell lymphoma 2
BCL-xL: B-cell lymphoma-extra large
BRD9: bromodomain-containing protein 9
BTK: Bruton’s tyrosine kinase
CDK4/6: cyclin dependent kinase 4/6
DC_50_: half-maximal degradation concentration
DLBCL: diffuse large B-cell lymphoma
DLT: dose limiting toxicities
EC_50_: half-maximal effective concentration
ER: estrogen receptor
HER2: human epidermal growth factor receptor 2
IKZF1/3: Ikaros family zinc finger protein 1/3
mCRPC: metastatic castration resistant prostate cancer
MTD: maximal tolerated dose
OS: overall survival
PFS: progression-free survival
PR: partial response
PROTACs: proteolysis targeting chimeras
PSA: prostate-specific antigen
VHL: von Hippel-Lindau

## Declarations

### Copyright

© The Author(s) 2021.
